# Supplementary figures and images for: Tissue specificity-aware TWAS (TSA-TWAS) framework identifies novel associations with metabolic, immunologic, and virologic traits in HIV-positive adults
Source: PLoS Genet. 2021 Apr 26;17(4):e1009464. doi: 10.1371/journal.pgen.1009464 (PMC8102009; doi:10.1371/journal.pgen.1009464)

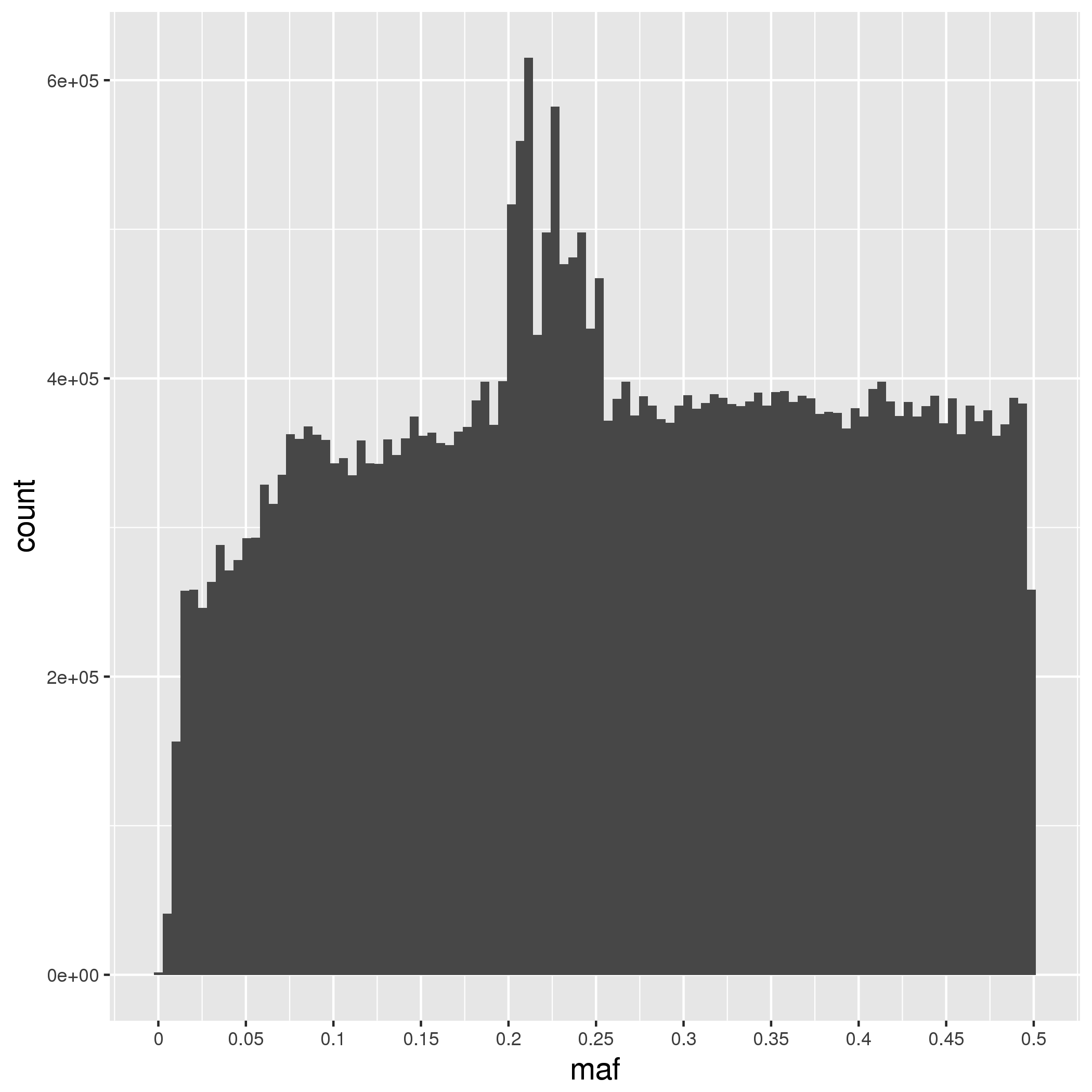

Supplement: S1 Fig — MAF of eQTLs closely resembled a uniform distribution, ranging between 1% to 50%, with a spike around 20–25%. (TIF) [file pgen.1009464.s001.tif]

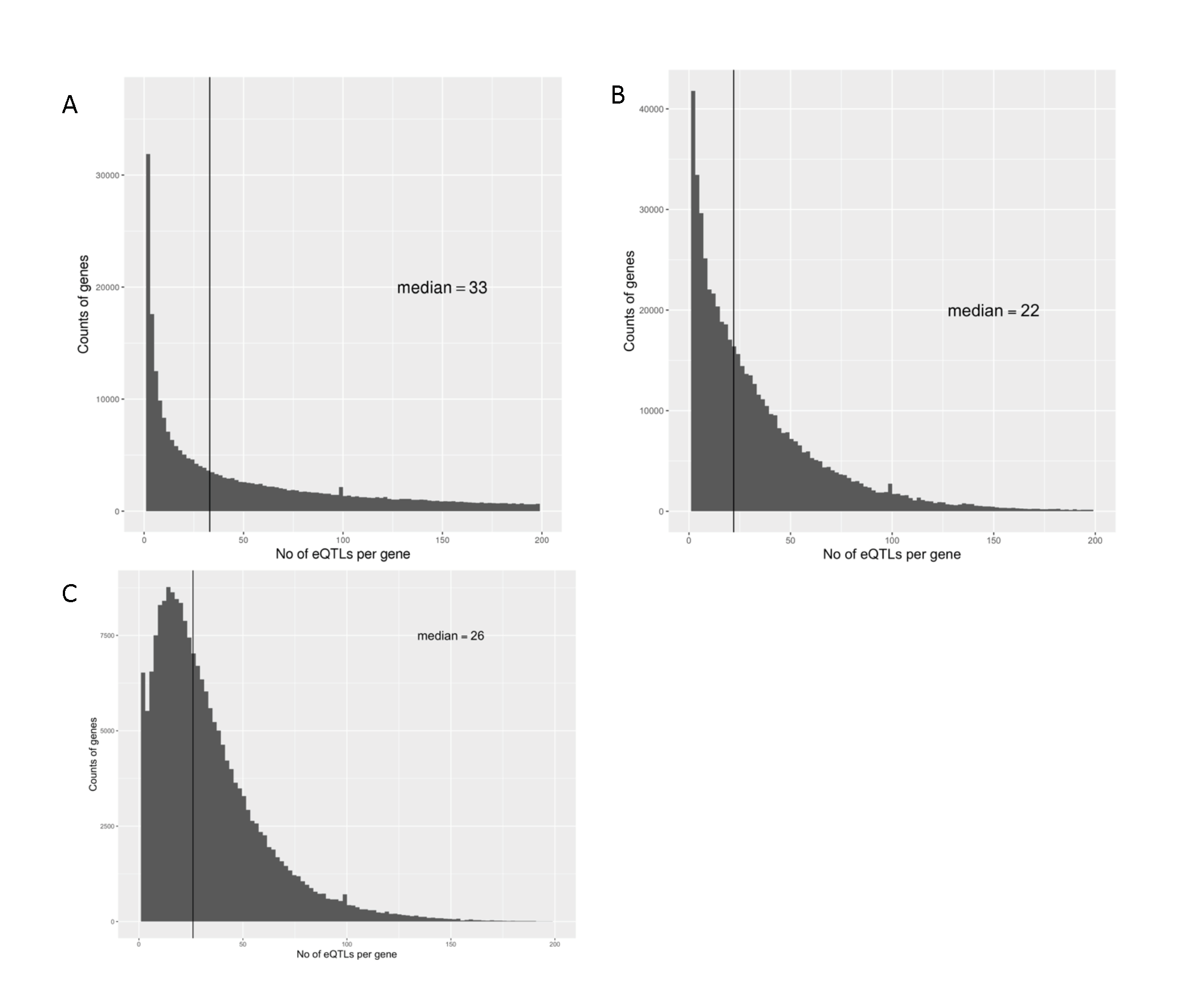

Supplement: S2 Fig — Distribution of number of eQTLs for a gene from (A) GTEx v7, (B) PredictDB eQTL datasets and (C) UTMOST eQTL datasets. (TIF) [file pgen.1009464.s002.tif]

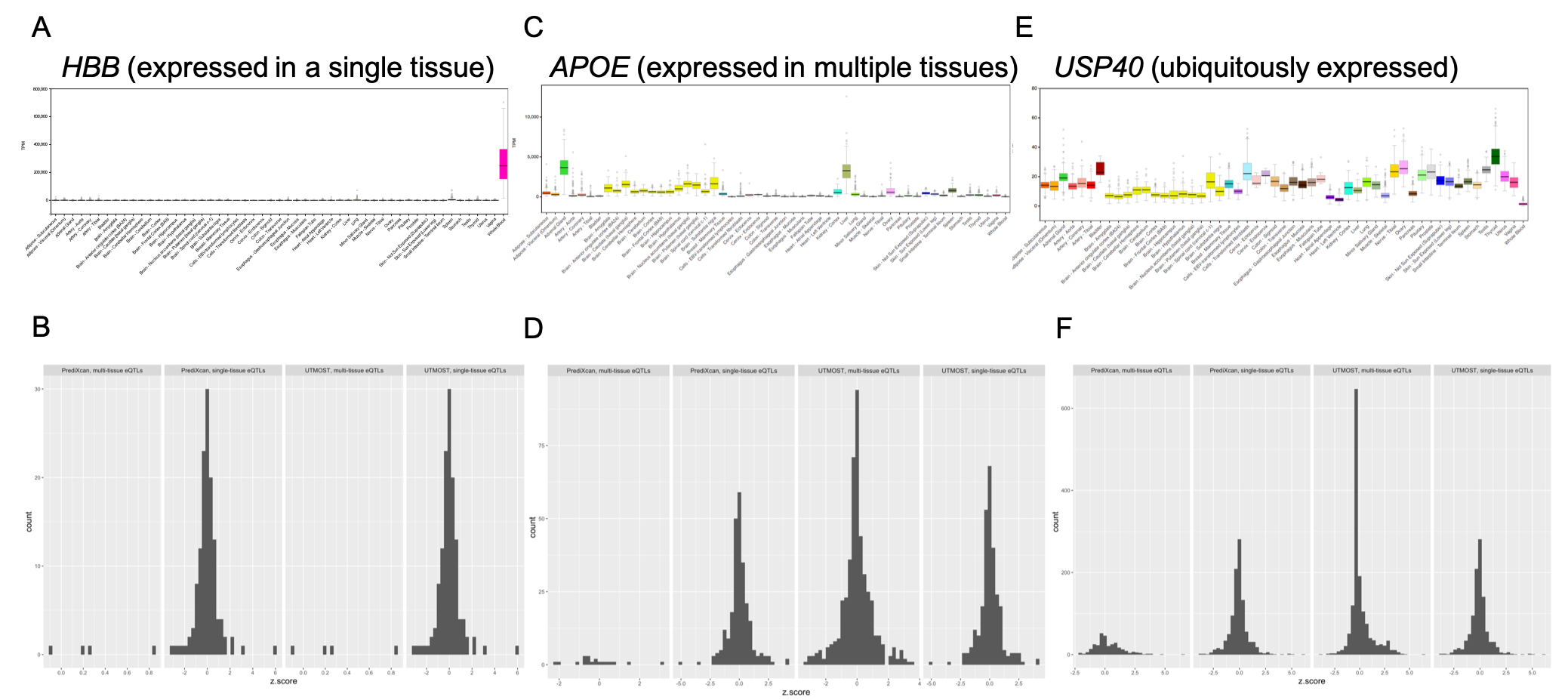

Supplement: S3 Fig — (A) and (B) For tissue-specific genes, like HBB, PrediXcan and UTMOST both identified eQTLs predominantly in a single tissue and eQTL weights followed a normal distribution. (C) and (D) For genes that are differentially expressed in multiple tissue, like APOE, PrediXcan and UTMOST both estimated normally distributed eQTLs weights. However, UTMOST was able to identify more eQTLs that are functioning across tissues. (E) and (F) For genes that are ubiquitously expressed in all tissues, like USP40, eQTL weights estimated from PrediXcan and UTMOST were both normally distributed. And again, UTMOST was able to identify more eQTLs that are effective in more than one tissues. (TIF) [file pgen.1009464.s003.tif]

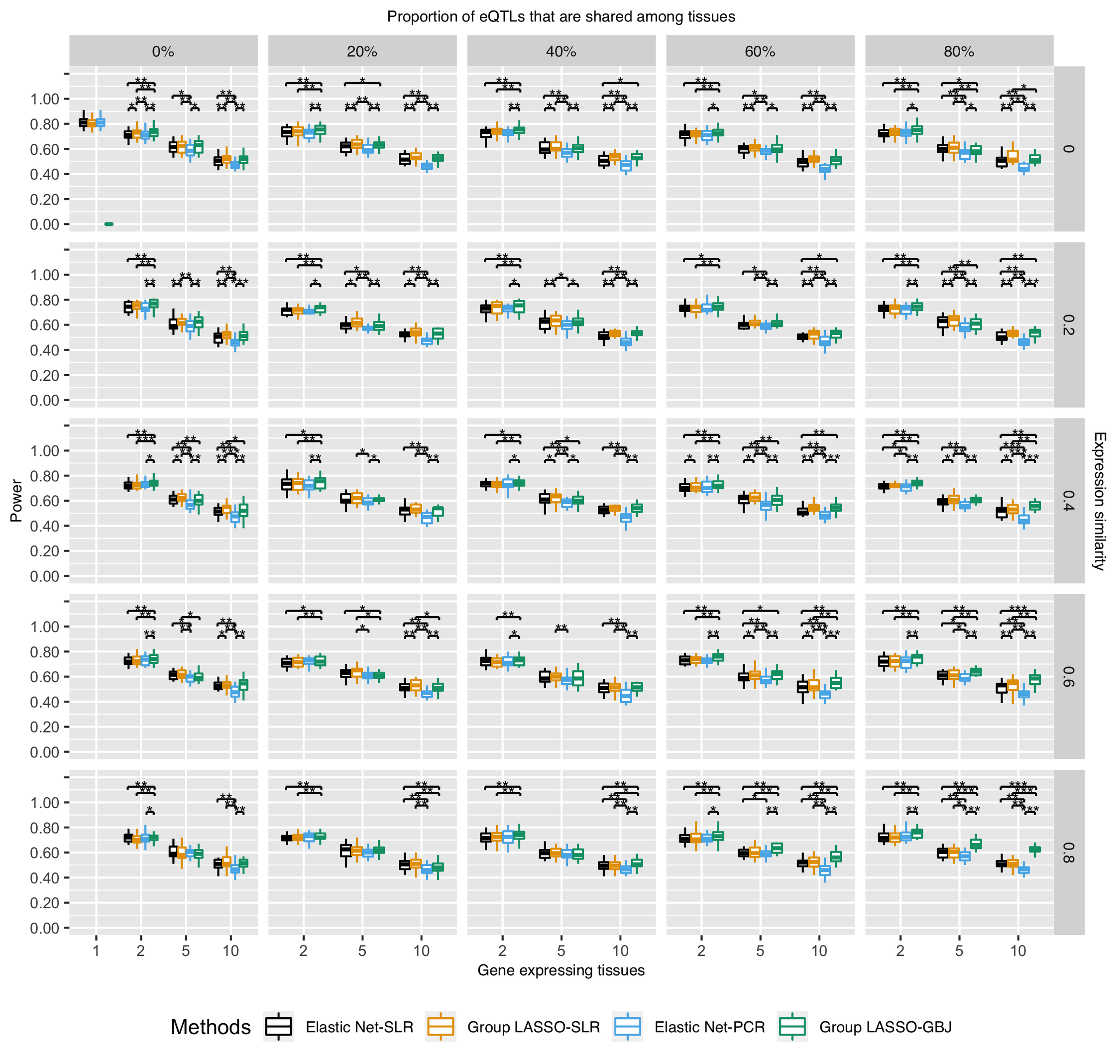

Supplement: S4 Fig — Power was the proportion of successfully prioritized gene-trait associations in the causal tissue out of all associations under the same simulation setting. X-axis is the number of gene-expressing tissues. Each column stands for the proportion of eQTLs that are shared among tissues for a gene. Each row is the similarity of gene expression profiles across tissues which is estimated by correlation. Moving from the top left to the bottom right is a gradient spectrum from tissue-specific genes to broadly expressed genes. The colors represent different TWAS methods and y-axis is the power. For tissue-specific genes at the top left, single-tissue TWAS (Elastic Net-SLR) and cross-tissue TWAS (Group LASSO-GBJ) had similar power. For broadly expressed genes at the bottom right, cross-tissue TWAS (Group LASSO-GBJ) had greater power. The difference in power among different TWAS methods were statistically evaluated (* p-value < 0.05, ** p-value < 0.01, *** p-value < 0.0001). (TIF) [file pgen.1009464.s004.tif]

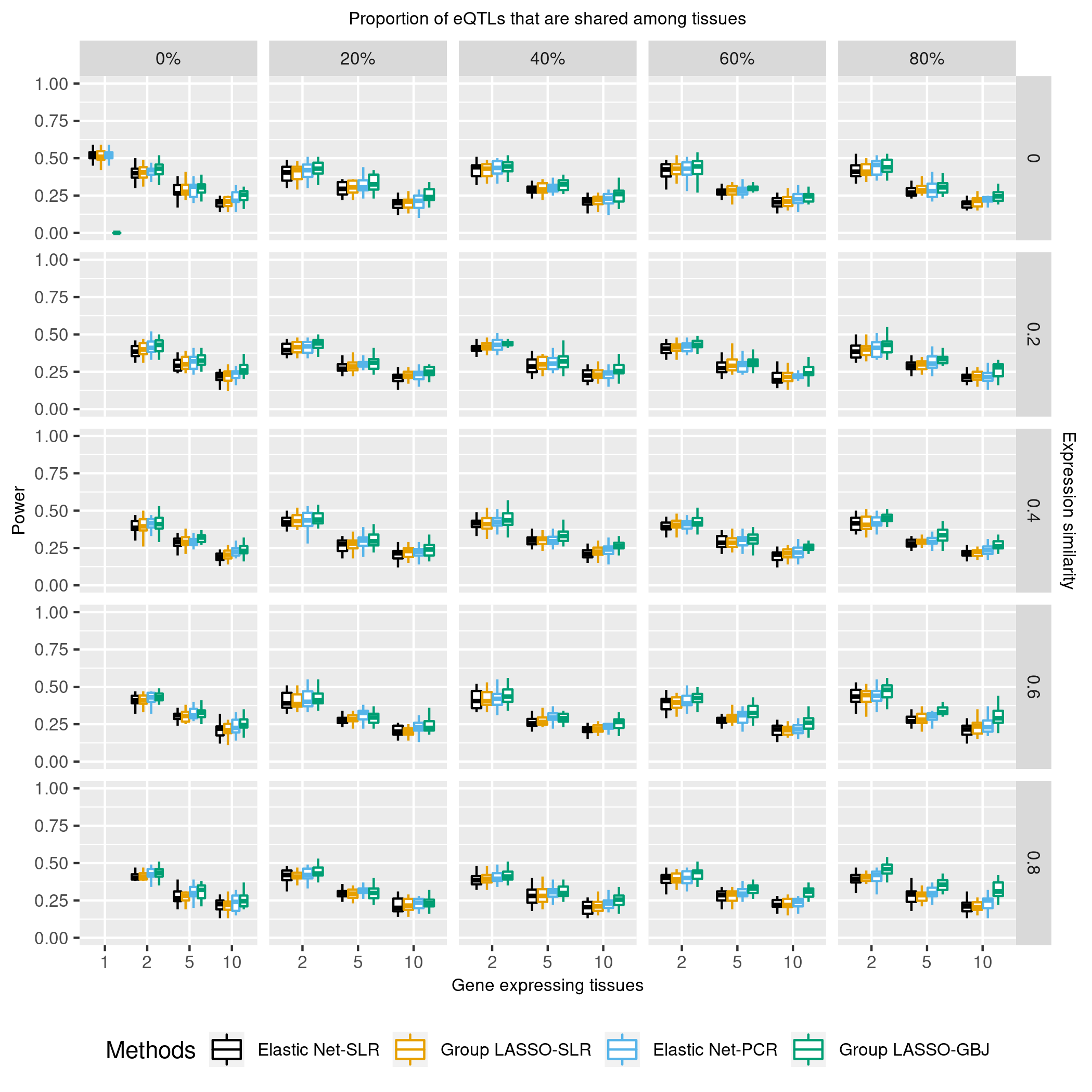

Supplement: S5 Fig — (TIF) [file pgen.1009464.s005.tif]

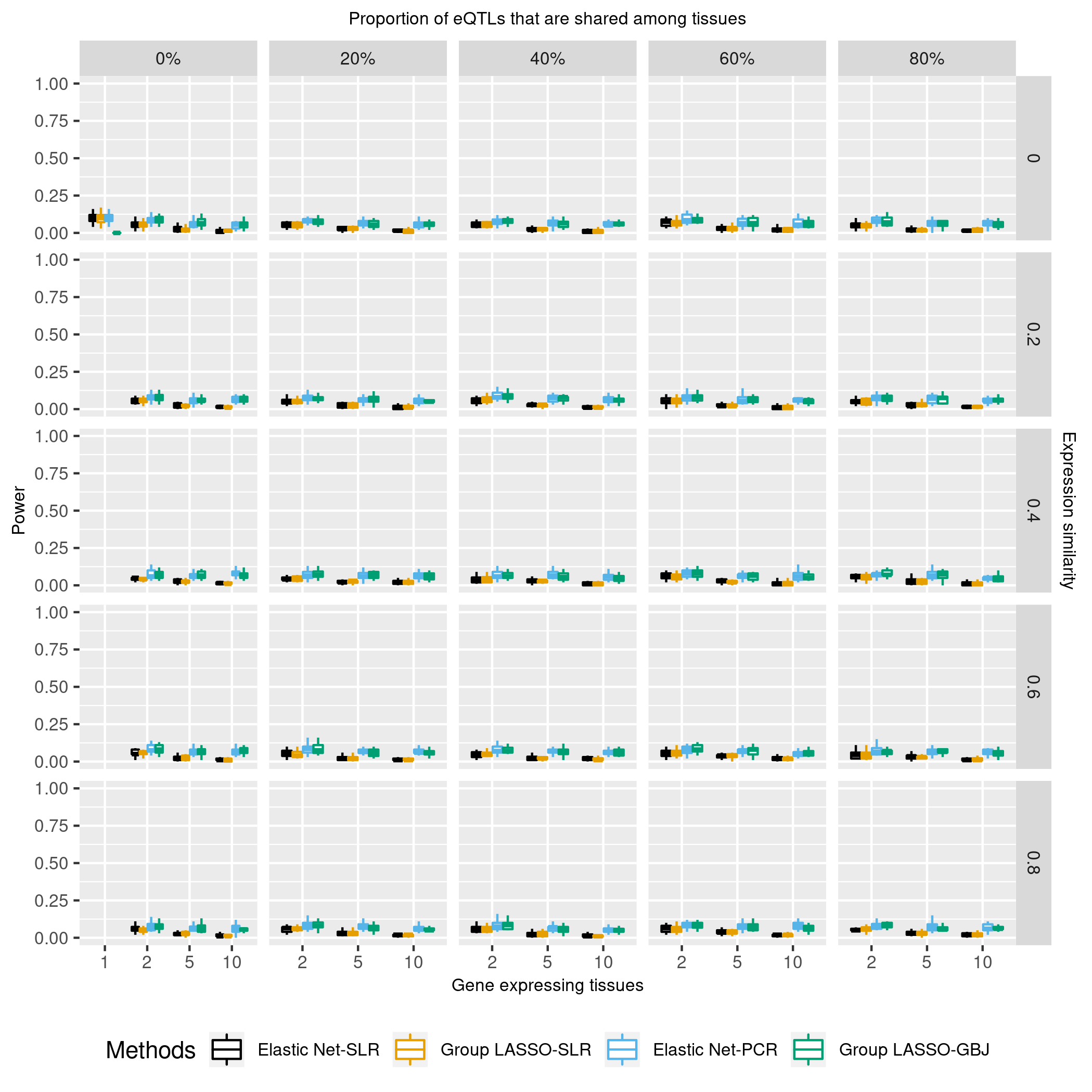

Supplement: S6 Fig — (TIF) [file pgen.1009464.s006.tif]

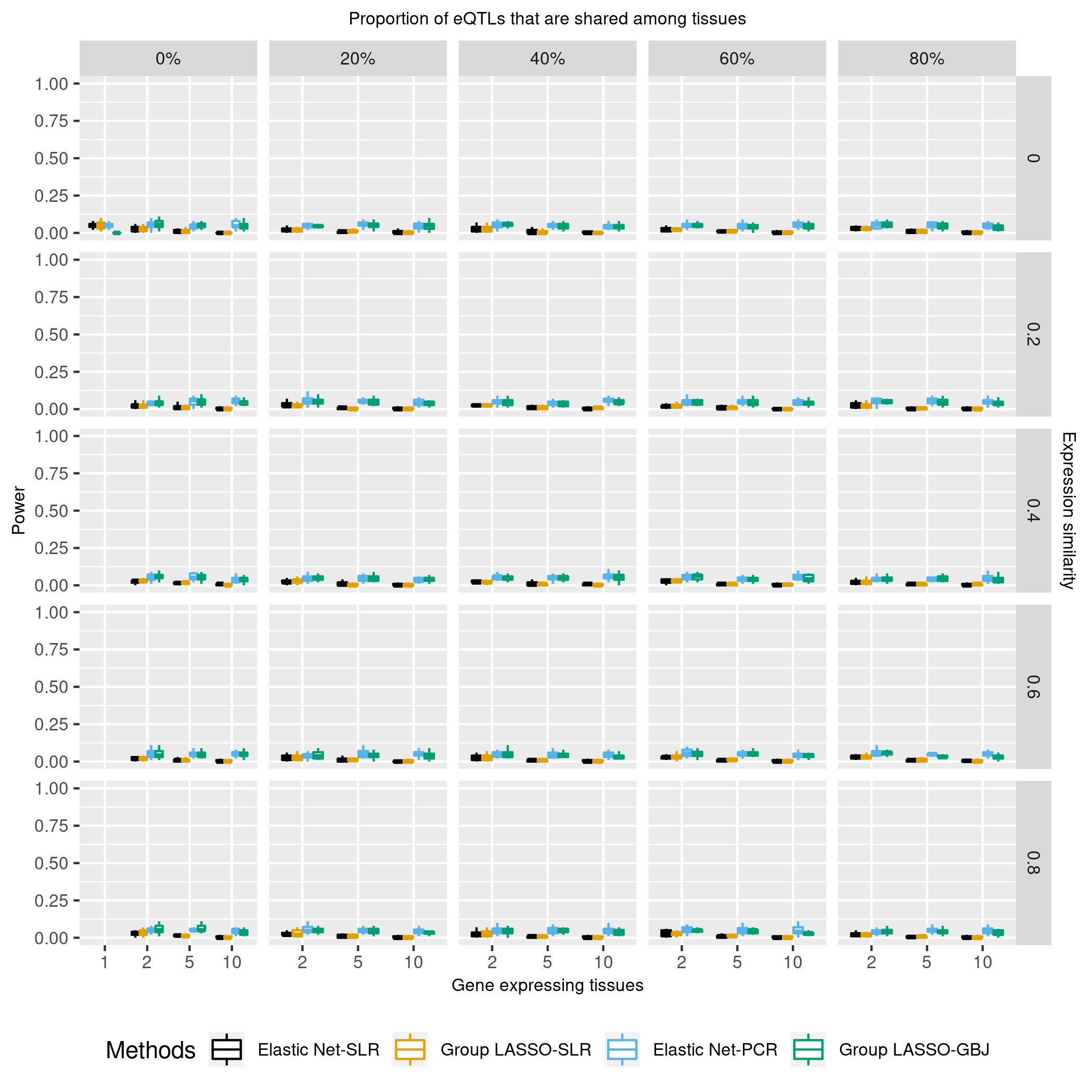

Supplement: S7 Fig — (TIF) [file pgen.1009464.s007.tif]

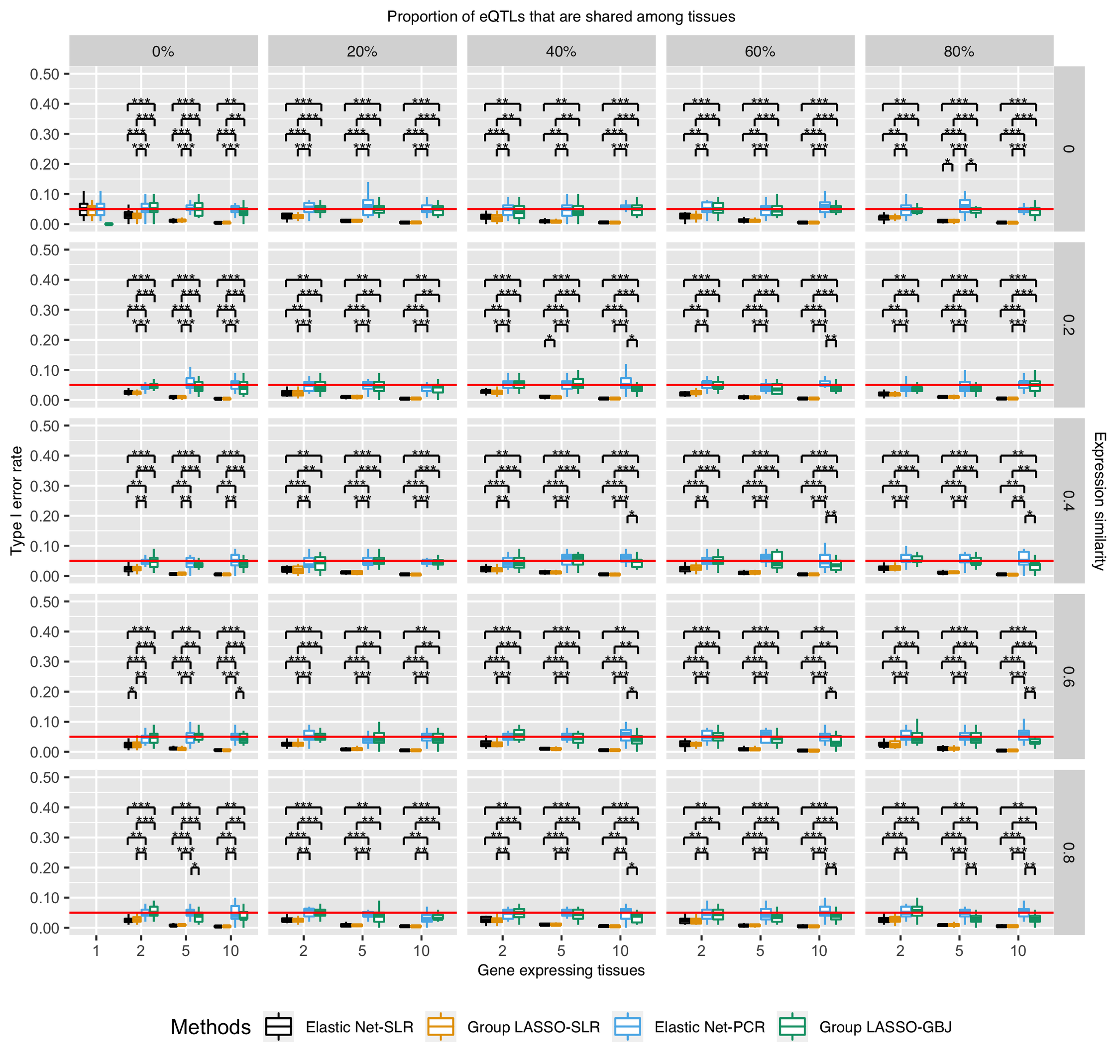

Supplement: S8 Fig — Type I error rate was the probability that TWAS wrongly identified a gene-trait association as significant while there was not any signal. Association p-values were controlled for the number of genes and tested tissues. X-axis is the number of gene-expressing tissues. Each column stands for the proportion of eQTLs that are shared among tissues for a gene. Each row is the similarity of gene expression profiles across tissues which is estimated by correlation. Moving from the top left to the bottom right is a gradient spectrum from tissue-specific genes to broadly expressed genes. The colors represent different TWAS methods and y-axis is the type I error rate. All TWAS methods had controlled type I error rates (≤ 5%). The difference in type I error rates among different TWAS methods were statistically evaluated (* p-value < 0.05, ** p-value < 0.01, *** p-value < 0.0001). (TIF) [file pgen.1009464.s008.tif]

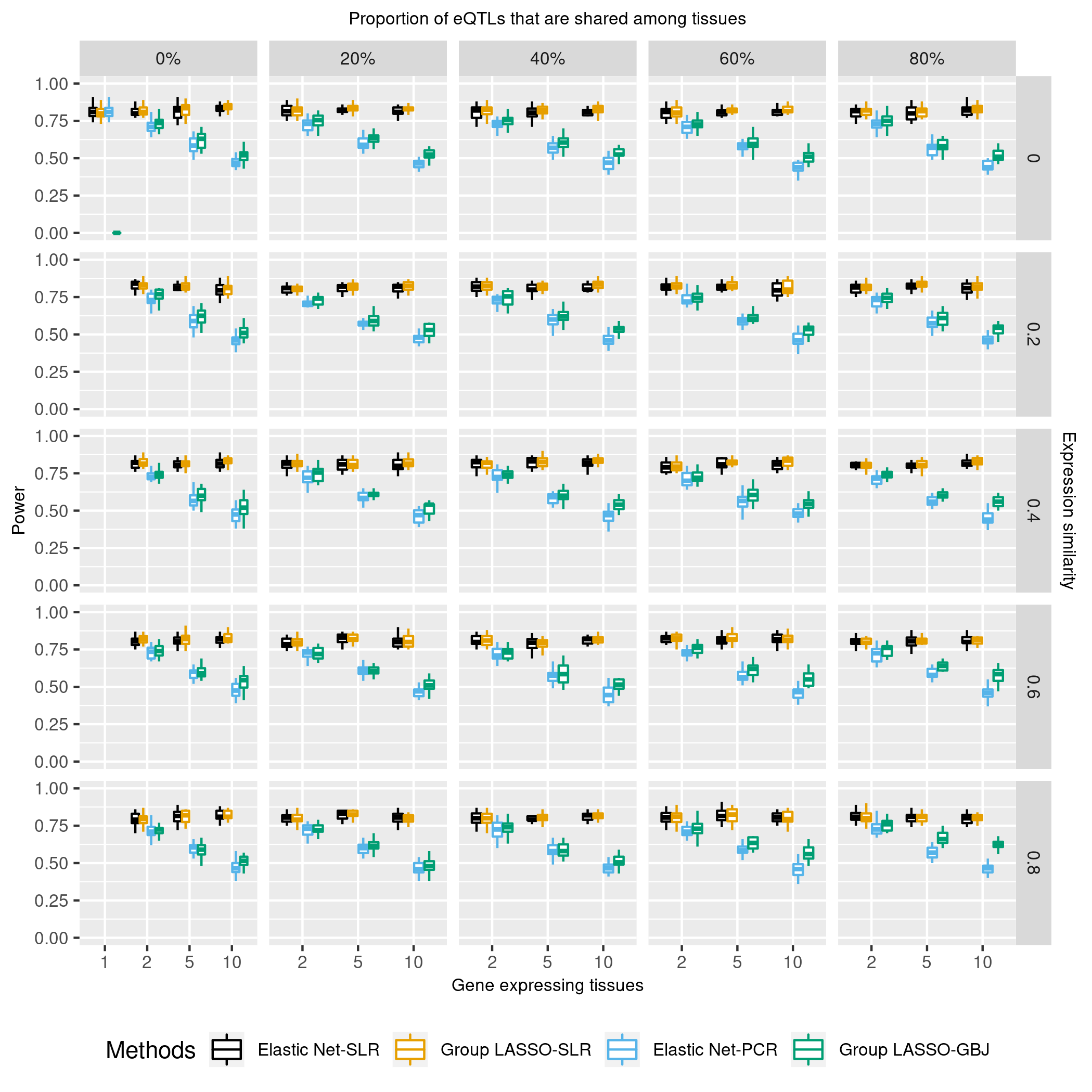

Supplement: S9 Fig — (TIF) [file pgen.1009464.s009.tif]

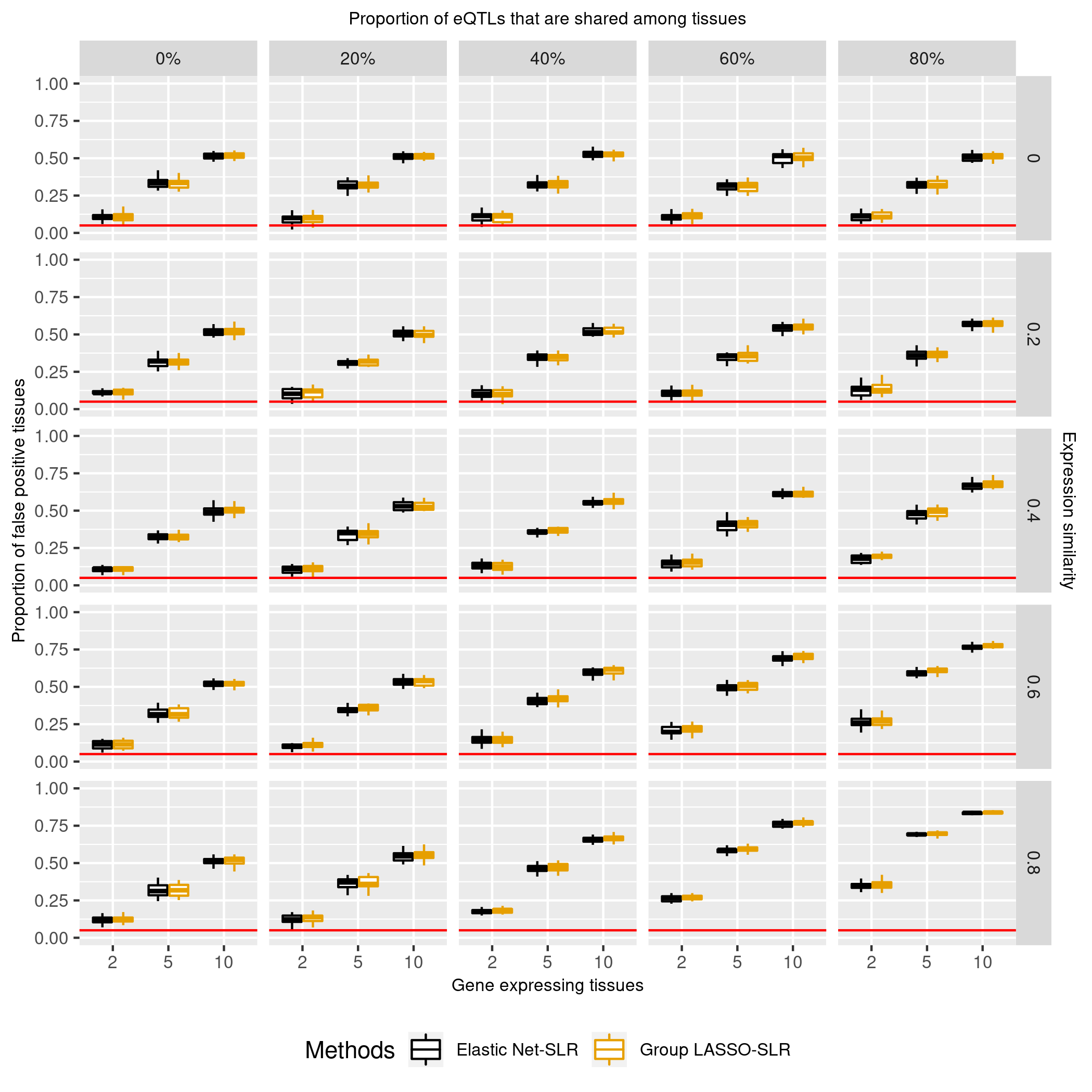

Supplement: S10 Fig — (TIF) [file pgen.1009464.s010.tif]

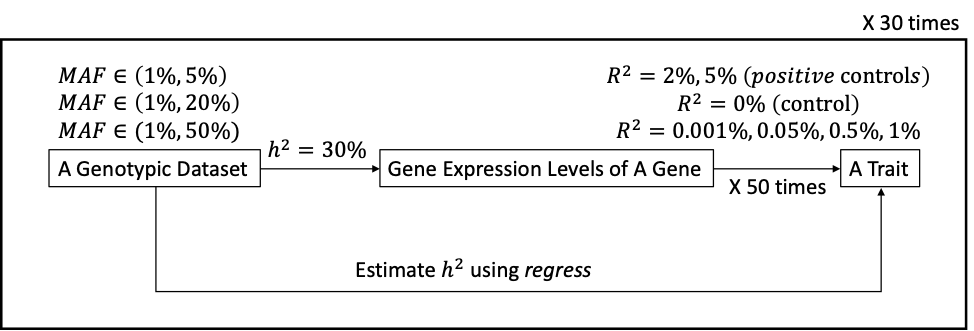

Supplement: S11 Fig — (TIF) [file pgen.1009464.s011.tif]

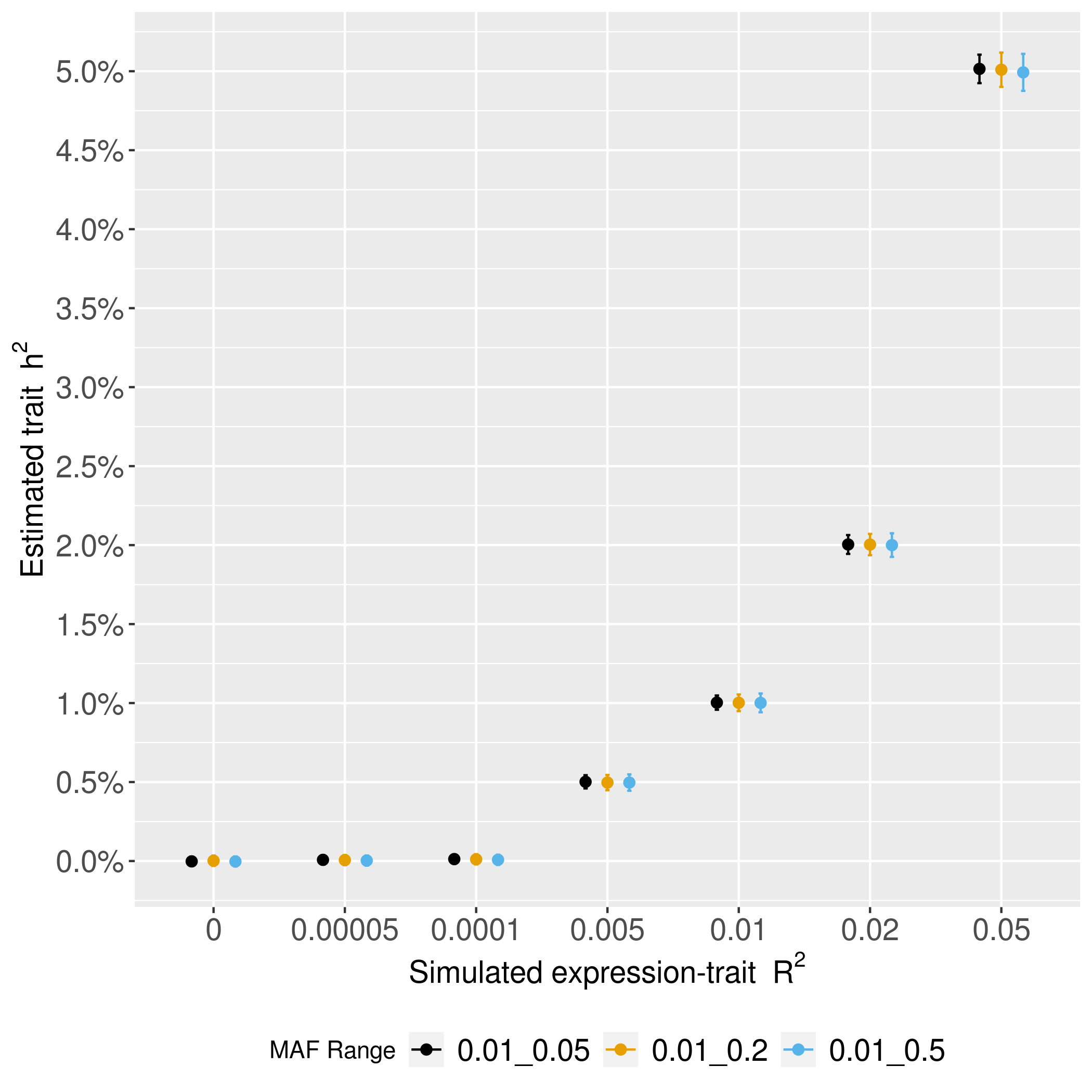

Supplement: S12 Fig — (TIF) [file pgen.1009464.s012.tif]

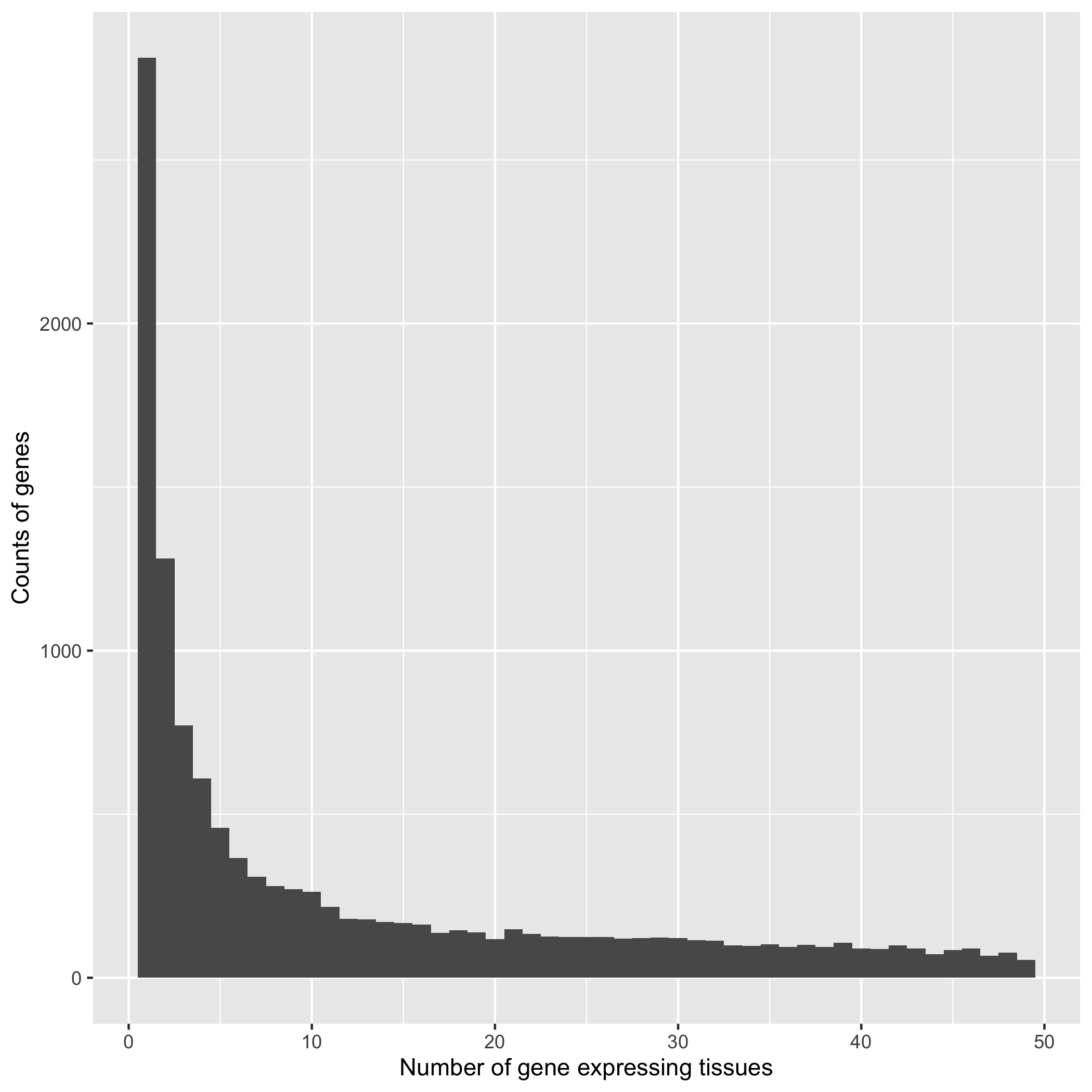

Supplement: S13 Fig — X-axis is the number of tissues that a gene was predicted to be expressed in based on GTEx v8 MASHR-based eQTL models. Y-axis is the count of genes. Genes tended to express in few numbers of tissue according to GTEx v8 prediction. (TIF) [file pgen.1009464.s013.tif]

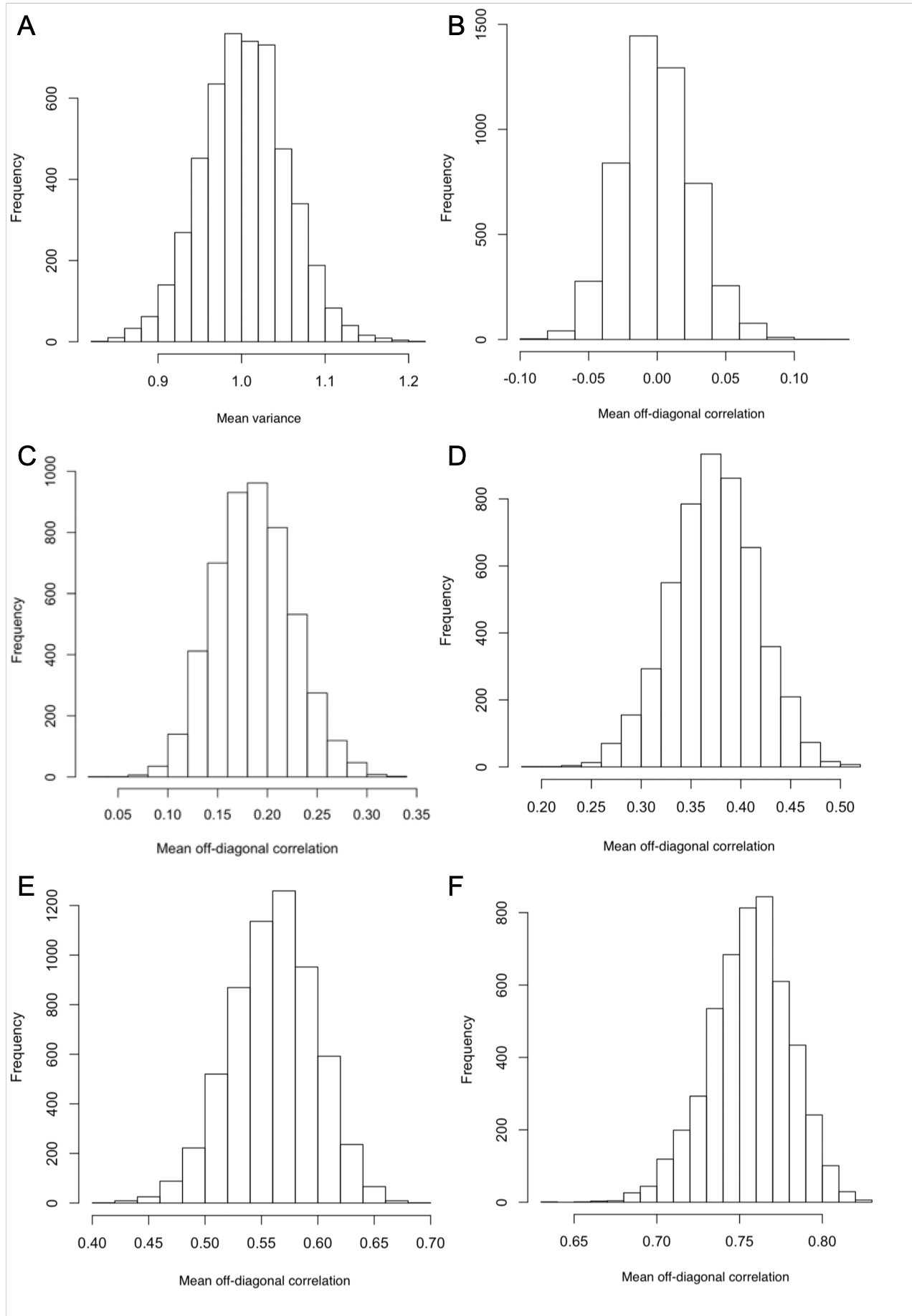

Supplement: S14 Fig — The number of tissues were five in this evaluation. Five thousand rounds of simulations were repeated for each value of cor(tissuep, tissuep′). In each repetition, we obtained one mean variance and one mean off-diagonal correlation across the five simulated tissues. (A) The empirical mean variance of EN×P were approximately one in any situations. The empirical mean off-diagonal correlations equaled 0, 0.186, 0.373, 0.563, and 0.756, for cor(tissuep, tissuep′) = 0 (B), 0.2 (C), 0.4(D), 0.6 (E), 0.8 (F). (TIFF) [file pgen.1009464.s014.tiff]

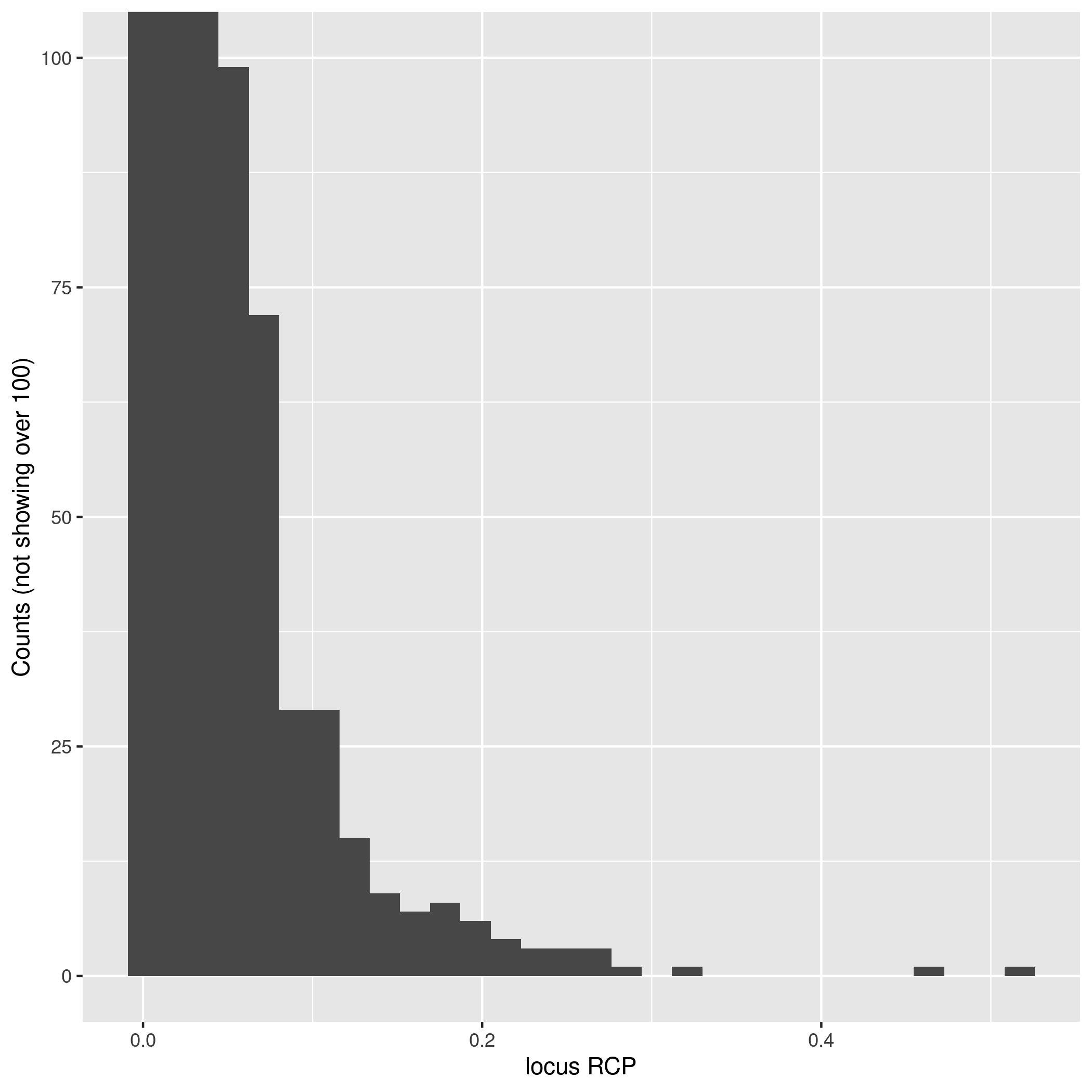

Supplement: S15 Fig — To evaluate for an appropriate locus RCP cutoff, we inspected locus RCP statistics for colocalization tests of 49 tissues, 10 statistically significant phenotypes, and ~20K genes. Approximately 10M were present in the figure. Y-axis were cut out at count of 100 for the ease of visualization. We took locus RCP > 0.025 as a cut-off based on the locus RCP distribution. (TIFF) [file pgen.1009464.s015.tiff]

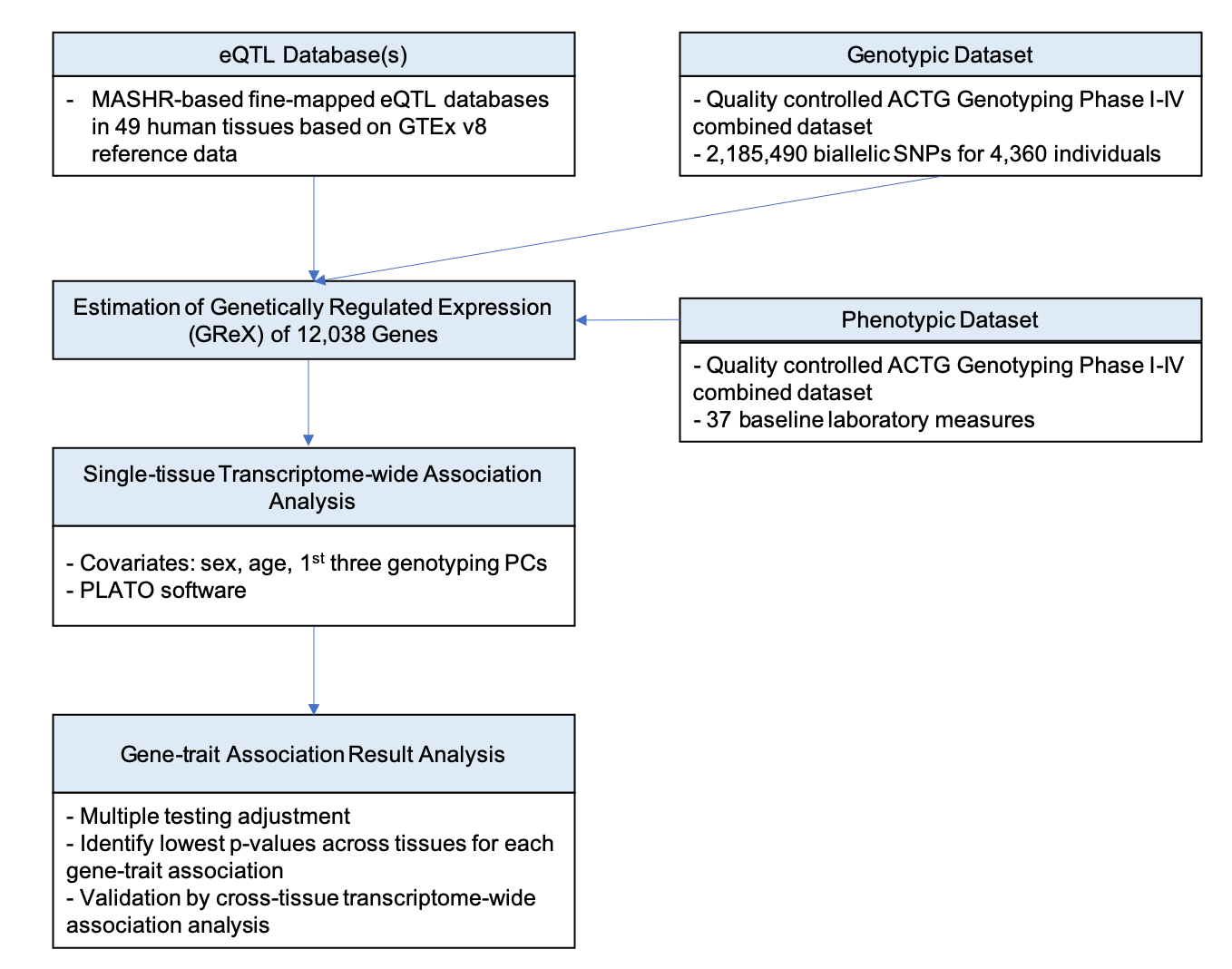

Supplement: S16 Fig — (TIF) [file pgen.1009464.s016.tif]

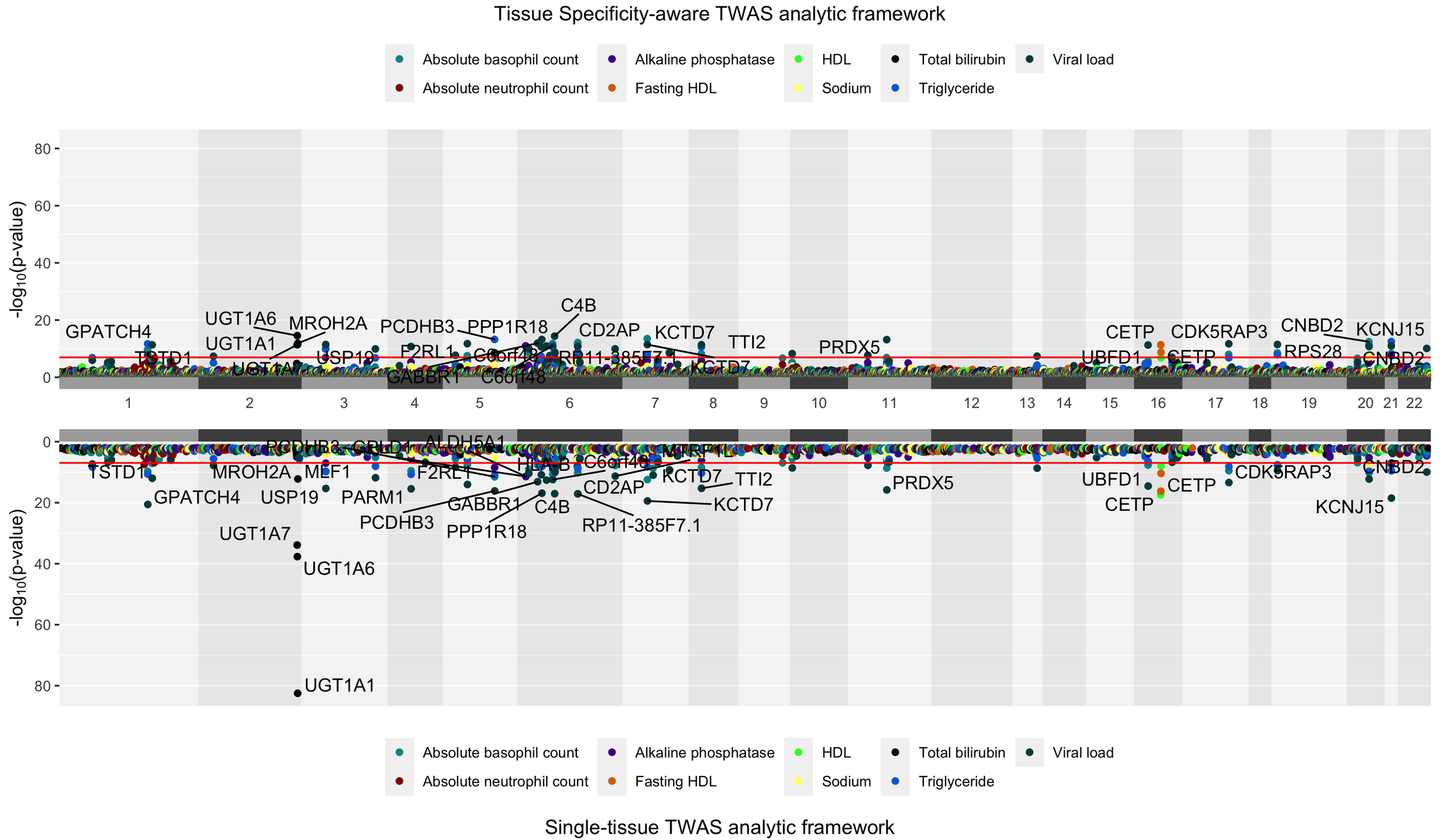

Supplement: S17 Fig — X-axis is the genomic location and y-axis is the -log10 transformed p-values. Colors denote different ACTG baseline laboratories. The significance threshold was 1.12×10^−7^ for both top and bottom TWAS frameworks. While single-tissue TWAS were able to find multiple significant gene-trait associations, the significant tissues did not necessarily connect to the traits of interest pathology-wise. Our tissue specificity-aware TWAS framework was able to retain all significant associations that were identified through regular single-tissue TWAS. (TIF) [file pgen.1009464.s017.tif]
