# Supplementary material for: Tissue specificity-aware TWAS (TSA-TWAS) framework identifies novel associations with metabolic, immunologic, and virologic traits in HIV-positive adults
Source: PLoS Genet. 2021 Apr 26;17(4):e1009464. doi: 10.1371/journal.pgen.1009464 (PMC8102009; doi:10.1371/journal.pgen.1009464)
Supplement: Attachment — Submitted filename: Review_Comments_PLOS_Genetics_v2.docx [file pgen.1009464.s021.docx]

December 17, 2020

Dear reviewers,

We hope that you enjoyed the holidays and are keeping a good spirit.

We want to thank you for the opportunity to revise our manuscript “*Tissue specificity-aware TWAS (TSA-TWAS) framework identifies novel associations with metabolic, immunologic, and virologic traits in HIV-positive adults*”. We appreciate your interest in the research topic and the constructive feedback of yours.

We believe that the manuscript is greatly improved after making the suggested edits. Following this letter are the comments in black with our responses in light blue. Thank you for your consideration.

Sincerely,

Marylyn D. Ritchie, PhD

Professor, Department of Genetics

Director, Center for Translational Bioinformatics, Institute of Biomedical Informatics

Associate Director for Bioinformatics, Institute of Biomedical Informatics

Associate Director, Center for Precision Medicine

University of Pennsylvania, Perelman School of Medicine

**Summary of revisions**

1. Clarified main text based on the reviewers’ comments
2. Clarified the discussions
3. Added an analysis and a figure that illustrated the power of TSA-TWAS
4. Added TWAS fine-mapping analyses using FOCUS to identify co-regulated genes near TSA-TWAS signals
5. Added a colocalization analysis using fastENLOC to identify colocalization signals for TSA-TWAS signals and fine-mapped co-regulated genes
6. Described the results and added discussions based on the additional analyses.

**Review Comments from PLOS Genetics**

Reviewer #1: Li et al. proposed a novel framework to integrate and maximize the performance of multiple TWAS methods.

1. In Figure 2, why the statistical power decreases with the gene expressing tissues increase? Do those genes have the same genetic heritability in different scenarios?

The statistical power decreased due to multiple testing burden that was imposed by increasing numbers of trait-irrelevant tissues. There was only one causal tissue across all of our simulated scenarios. But the true gene-trait signals in the causal tissue were diluted by the noisy association tests performed in irrelevant tissues. Therefore, the power decreases while the gene expression increases.

It was analogous to real-world scenarios where we don’t necessarily know the causal tissues for certain complex traits or diseases. One will choose to test for association signals across all available tissues to see if any genes are detected as statistically significant. This design, however, as indicated in our simulation study, faces the challenge of multiple testing burden.

Yes, all genes had the same genetic heritability in different scenarios.

2. In line 265-266, the authors claimed that “if trait-related tissue(s) are uncertain, it may be better to stratify genes based on the number of tissues in which the genes are predicted to be expressed”, but it is not clear how the number of tissues can affect the choice of TWAS frameworks.

Thanks for pointing this out. We clarified the sentence. As tissue-specificity of genes affect the choice of TWAS methods, we meant to differentiate analyses for tissue-specific genes and genes that are ubiquitously expressed in multiple tissues.

3. The authors claimed that the novel framework considers the potential false positive rates of tissue identifications. In real-data analysis of 37 baseline laboratory values, authors didn’t show the tissue-trait association results. At least, for those identified trait-associated genes, it is not clear in which tissues those genes were identified.

Sorry for the confusion. You pointed out one disadvantage of cross-tissue TWAS methods. Cross-tissue TWAS is an integrative analysis that combines associations across all tissues. The cross-tissue gene-trait association results indicate if a gene is statistically significant in at least one tissue, but the results do not indicate which tissues the gene is significant in.

In our real-data analysis in the ACTG data, all of our statistically significant results were from cross-tissue TWAS. Thus, no specific tissue information was available for any of the results.

We made up for the disadvantage of cross-tissue TWAS by performing a colocalization analysis for statistically significant genes. Colocalization analysis implicated whether the association signals colocalize with eQTL signals in specific tissues. But we warn that colocalization can be conservative as well.

4. In line 323 and 324, the authors claimed that some of those pleotropic genes were located on the MHC region of chromosome 6, while the MHC region has a rather complicated LD structure and thus those identified results could possibly be false positives. However, the authors didn’t discuss about this point.

We acknowledge this discovery in our discussion, but sincerely defer this research question to future research. Answering this question will require specific genotyping/sequencing of the MHC region.

5. In line 431, “Genotypes were simulated as biallelic SNPs”, were LD structures considered when simulating genotypes?

No, we did not simulate LD structures, but simulated independent eQTLs. For TWAS that use individual-level genotype data (e.g. PrediXcan), One can consider our simulation study as an ideal case where an independent eQTL represented effects of multiple high-LD eQTLs which can be observed in natural biological data.

We do recognize that in real-data analyses, eQTLs can be in LD with one another and not all eQTLs in LD may be present in the genotype data. The solutions to the issue depend on the eQTL models that one chooses to use:

1. In our ACTG real-data analysis, we used MASHR-based eQTL models that were generated from GTEx v8 reference panel.
   a. Note, MASHR-based eQTL models include fine-mapped eQTLs. The underlying method picks ONE top variant from an LD cluster. Thus, most eQTLs are in low LD with each other. (<https://groups.google.com/g/predixcanmetaxcan/c/ksmvtcEBMWc/m/QYt8eCxUAwAJ>):
   b. Since MASHR-based eQTLs are in low LD with each other, we did not go back to simulate LD. We wanted our simulations to mirror our real data analysis in this way.
2. If one is using elastic net-based eQTL models, there are likely several eQTLs that are in LD with each other. One’s best course of action is to impute genotype data to a densely genotyped reference panel. Moreover, elastic net-based eQTL models should be more robust than, say, a LASSO-based eQTL model to missing SNPs. (<https://groups.google.com/g/predixcanmetaxcan/c/IFfsea1lF84/m/bJf_0Ru9AQAJ>)

6. To show the power/false discovery rate of the proposed framework, it will be better if the author can conduct simulation analysis using the proposed framework or at least summarize the potential performance of the proposed framework based on the current simulation results.

Thanks for the suggestion. We agree that this would be a useful simulation and thus have added that to the manuscript. We summarized the TSA-TWAS power in the new figure 6.

Reviewer #2: Binglan Li and colleagues present a comprehensive evaluation of single-tissue and cross-tissue transcriptome-wide association study methods using simulations with a focus on two methods PrediXcan and UTMOST. They develop a tissue-specificity aware analytic framework and apply their results GWAS data for laboratory traits from AIDS clinical trials. Overall this is an important methodological contribution to the growing field of transcriptome-wide association studies that helps further clarify the role of tissue specificity and cross-tissue and pleiotropic effects in influencing TWAS results. I do however have a few concerns and these are elaborated on below.

Major comments:

In their application of the TSA-TWAS framework to the ACTG data set, the authors identify several gene expression-trait associations where for the same trait multiple genes in the same genomic region are implicated. For example, UGT1A6-MROH2A-UGT1A1-UGT1A7 and total bilirubin in Table 3 and CD2AP and RP11-385F7.1 and absolute basophil count in Table 4, to name a couple of such instances. The current implementations of PrediXcan and its variations recommend coupling the analysis with enloc (reference 57 in this manuscript by Li et al). Why do the authors choose not to evaluate colocalisation between the GWAS signal and the eQTL signal to potentially filter associations at such multi-gene association regions?

Thanks for the suggestion. Our analyses were performed before the PhenomeXcan paper was published, which is why colocalization analysis wasn’t there in the first submission. But we agree that colocalization is an important approach to support our ACTG discoveries.

In this revision, we have added colocalization analyses results to tables 3 and 4. Interestingly, only UGT1A1 of the UGT1A6-MROH2A-UGT1A1-UGT1A7 region was supported by the colocalization result. However, colocalization is a conservative approach and is influenced by different LD structures between eQTL reference panel and our ACTG cohort. The associations that lacked colocalized signals should not necessarily be interpreted as false positives, but simply, no independent analytic evidence to supports the discovery.

Page 26, line 515, under Phenotype: How was linkage disequilibrium (LD) structure modelled, if at all, in the GWAS data set for the Phenotype in the simulations? There seem to be two forms of correlations that affect TWAS results that aren't clearly addressed in the simulation -- (1) correlation between SNPs due to LD in the phenotype or trait GWAS and (2) expression correlation between genes that are located physically near each other on the genome (a known phenomenon, see for example, Michalak, Genomics, Volume 91, Issue 3, March 2008, Pages 243-248). The authors highlight UGT1A1 as the target gene in the Abstract but rightly suggest the potential for multi-gene involvement on page 19, line 363 but this presents a paradox that is not addressed. What is the evidence from GWAS that there is a single causal gene at a locus or multiple causal genes at a locus for complex traits? How is this accounted for in TSA-TWAS and its underlying simulation framework? How does this affect the power of the approaches?

Many important questions were asked here.

If I understood the first question correctly, the reviewer was interested in pleiotropy and how we simulated effects of pleiotropic loci for phenotypes. Even though our real-world ACTG analysis indeed found pleiotropic effects, our simulation focused solely on the influence of tissue-specificity of genes on TWAS performance. We assumed that there was only one causal tissue. This causal tissue determined one and the only one phenotype. If a gene was simulated to be expressed in multiple tissues, all the rest of the tissues were noise and did not contribute to the phenotype. We do see the potential use of this simulation scheme for a pleiotropy-specific simulation study, e.g. replacing simulation of tissues with phenotypes. We look forward to any opportunity of collaborations.

The second question asked how the study addressed the co-expression of neighboring genes and how to identify if there are one or more possible causal genes. Thank you for providing an interesting paper. We have cited the work and added a discussion of how neighboring genes that are either co-expressed or correlated in LD likely impact TWAS performance. We also added a discussion of how to deal with the issue in the discussion section.

The second question was discussed in Wainberg *et al. Nature Genetics* 2019. Genes can have correlated gene expression levels due to either co-regulation or high LD among eQTLs. These genes tend to be picked up together by TWAS as shown for the UGT1A1 2q37.1 locus (we clarified the argument in the discussion).

To address the second question in real-data analyses, we followed the best practice suggested in the *Wainberg et al.* We added a TWAS fine-mapping analysis (FOCUS by Mancuso *et al. Nature Genetics* 2019) to identify an additional set of likely causal genes that neighbored TWAS signals based on LD among eQTLs.

Noteworthy, we used the multi-panel FOCUS eQTL database that combines PrediXcan GTEx v7 weights with other eQTL weights from FUSION (Gusev *et al.* Nature Genetics 2016). The reason of using GTEx v7 instead of latest GTEx v8 eQTLs was the technical difficulty of preparing GTEx v8 weight database for FOCUS. The issue has been reported to the FOCUS group over the course of the last half a year (issue 25 and 26 on the FOCUS GitHub issue: <https://github.com/bogdanlab/focus/issues/25>, <https://github.com/bogdanlab/focus/issues/26>).

To identify if there were a single gene or multiple trait-related genes at a risk locus, we performed colocalization analyses on all fine-mapped genes. Colocalization provides additional molecular mechanistic evidence of possible transcriptional impact on a phenotype. Due to the conservative nature of colocalization analyses (expecting the same population structure and LD structure between the test and eQTL cohorts), not many genes were supported by colocalization signals.

To sum up, co-expression of neighboring genes is a confounding factor in TWAS. It poses technical difficulties that cannot be solved by GWAS and gene expression data alone. We performed additional TWAS fine-mapping and colocalization analyses to identify co-regulated neighboring genes and which of them are more likely to be transcriptionally regulated. Further research is expected to be done in this area of research, for example, greater sample sizes of reference transcriptome data to improve eQTL accuracy, incorporation of functional genomics data, and high-throughput reporter assay for validations.

Page 18, line 318: are the pleiotropic genes expressed across tissues or are they tissue specific?

All pleiotropic genes that we identified as statistically significant were expressed in more than one tissue.

Minor comment:
Page 15, lines 311 to 313 (ATF6B...) - "For instance, ATF6B is a ... and it has been associated
with HIV-associated neurocognitive disorders in previous research." - please provide a citation to support this statement.

Thank you for catching this. We added the reference.
