# Supplementary material for: Tissue specificity-aware TWAS (TSA-TWAS) framework identifies novel associations with metabolic, immunologic, and virologic traits in HIV-positive adults
Source: PLoS Genet. 2021 Apr 26;17(4):e1009464. doi: 10.1371/journal.pgen.1009464 (PMC8102009; doi:10.1371/journal.pgen.1009464)
Supplement: Attachment — Submitted filename: Review_Comments_PLOS_Genetics_2nd_Revision_v2.docx [file pgen.1009464.s022.docx]

February 5, 2021

Dear reviewers,

We hope that you are having a good start of a new year.

Again, we want to thank you for the opportunity to continue to revise our manuscript “*Tissue specificity-aware TWAS (TSA-TWAS) framework identifies novel associations with metabolic, immunologic, and virologic traits in HIV-positive adults*”.

Following this letter are the comments in black with our responses in light blue. Thank you for your consideration.

Sincerely,

Marylyn D. Ritchie, PhD

Professor, Department of Genetics

Director, Center for Translational Bioinformatics, Institute of Biomedical Informatics

Associate Director for Bioinformatics, Institute of Biomedical Informatics

Associate Director, Center for Precision Medicine

University of Pennsylvania, Perelman School of Medicine

**Summary of revisions**

1. Clarified main text (lines 171-176 and lines 286-289) based on the reviewer’ comments

**Review Comments from PLOS Genetics**

Reviewer #1:

1. In responses to “In Figure 2, why the statistical power decreases with the gene expressing tissues increase? Do those genes have the same genetic heritability in different scenarios?”, the author claimed that the decreasing power is due to the increased number of tests, but it shouldn’t be case for the Group Lasso-GBJ since it is a burden test considering all tissues together. This figure is also contradictory to Line 173-175

Yes, Multiple testing burden affected the power of TWAS to identify trait-related genes. This is also true for the Group Lasso-GBJ. The reviewer is right that the Group Lasso-GBJ considers all tissue together. Taking a step back, the Group Lasso-GBJ integrates association statistics for each single tested tissue and performs a secondary statistical evaluation.

The secondary statistical test by the GBJ has naturally taken the number of tissues into consideration when evaluating statistical significance (<https://doi.org/10.1007/BF00533250>). The feature of the GBJ test is that it accounts for correlated gene expression levels while other methods treat each tissue independently (<https://doi.org/10.1080/01621459.2019.1660170>), which is the reason that the Group Lasso-GBJ had a better performance for identify genes that are similarly expressed in multiple tissues.

We do see how the lines 173-175 may be confusing as previously written and thus, we have clarified these lines in the main text. The lines 173-175 were intended to explain how the tissue specificity of eQTLs, rather than the number of tissues, affected TWAS performance. And it only focused on the Group Lasso-GBJ test and how the method had greater power to prioritize genes when, and only when, the similar expression levels of genes across tissues were driven by an underlying genetic force (multi-tissue eQTLs).

2. In Figure 6, Group-Lasso GBJ showed comparably lower power than other methods when a higher percentage of genes are tissue-specific genes, while in Fig 2, all methods have lower power for genes expressed in multiple tissues. It is understandable for Group-Lasso GBJ to have higher type-I error rates for tissue-specific genes, but it will be better to discuss about the lower power of Group-Lasso GBJ method

We added the discussion about the lower power of Group-Lasso GBJ method at line 215-216. The issue pertains to how the GBJ test was designed and was first mentioned at line 203-215. Additionally, Fig 2 evaluated tissue-specific genes and genes that were expressed in multiple tissues, separately. Fig 6 combined those two kinds of genes together to evaluate the power of a TWAS method or analytic framework. By using the TSA-TWAS framework, the optimal method (Elastic Net-SLR) is used on the genes expressed in a single tissue while simultaneously, the optimal method (Group Lasso-GBJ) is used on the genes expressed in multiple tissues.

Reviewer #2:

Thank you again for the helpful feedback.
